# Assessment of Pre-analytical Errors and Fostering Strategies to Enhance Accurate Results and Efficient Turnaround Times in the Cytology Laboratory of a Tertiary Care Hospital

**DOI:** 10.7759/cureus.56592

**Published:** 2024-03-20

**Authors:** Vallal Kani, Kavitha K, Sulochana Sonti

**Affiliations:** 1 Department of Pathology, Saveetha Medical College and Hospitals, Saveetha Institute of Medical and Technical Sciences, Saveetha University, Chennai, IND

**Keywords:** quality control, cytology, turnaround time, sample rejection, pre-analytical errors

## Abstract

Introduction

Pre-analytical errors in cytology laboratories can significantly impact the accuracy of diagnostic results and turnaround times, ultimately affecting patient care. This article presents an evaluation of pre-analytical errors and proposes fostering strategies to enhance accuracy and efficiency in the cytology laboratory of a tertiary care hospital. The background discusses the importance of pre-analytical processes in ensuring reliable cytological diagnoses and the common errors encountered in specimen collection, handling, and transportation. Strategies for error reduction and improvement in turnaround times include staff education, standardization of procedures, utilization of appropriate collection and transport devices, implementation of quality control measures, and utilization of automation technologies. By addressing pre-analytical errors and implementing fostering strategies, cytology laboratories can optimize diagnostic accuracy, improve patient care outcomes, and enhance overall laboratory efficiency.

Aims and objectives

This study aims to assess the prevalence and nature of pre-analytical errors in the cytology laboratory of a tertiary care hospital to understand the extent of the issue, identify the specific factors contributing to pre-analytical errors like specimen collection, handling, and transportation processes, and pinpoint areas for improvement. It seeks to evaluate the impact of pre-analytical errors on the accuracy of cytological results and the efficiency of turnaround times, highlighting the consequences for patient care. Furthermore, the study aims to develop targeted strategies to minimize pre-analytical errors and enhance the accuracy of cytological results.

Materials and methods

This study was conducted at the Cytology Laboratory of our hospital from January 2023 to December 2023 after getting proper approval from the Institutional Review Board (IRB approval number 101/02/2024/PG/SRB/SMCH). It is a retrospective analytical study, and a total of 5412 samples from patients of the outpatient (OP) department, inpatient (IP) department, and community health outreach program facilities received in the cytology laboratory were analyzed during the study period. The inclusion criteria were the test samples sent specifically for cytological analysis. The samples sent for biochemical or microbiological examination were excluded. The frequency of sample distribution and rejected samples were calculated and the results were correlated.

Results

A total of 5,412 samples received in the cytology laboratory were analyzed during the study period. The majority of the samples were Papanicolaou smears (2,352, 43.5%), followed by fluid cytology (1,008, 18.6%) and ultrasound-guided fine-needle aspiration cytology (FNAC, 984, 18.2%). Of the total number of samples, 225 (4.16%) were repeated and 27 (0.5%) were rejected.

Conclusions

Pre-analytical, analytical, and post-analytical processes are the three key factors that determine the dependability and precision of cytological test results. Detecting critical alerts such as the positivity of malignancy underscores the paramount importance of result accuracy. Implementing good laboratory practices and conducting both external and internal audits can reduce the frequency of preventable errors in a cytology laboratory, thereby ensuring enhanced precision and expedited outputs.

## Introduction

Laboratory results play a crucial role in identifying and organizing disease types, comprehending their clinical implications, and devising treatment strategies. Effective total quality management is essential for laboratories to uphold standards, minimize errors, and enhance patient well-being. This approach encompasses all phases of laboratory processes, including pre-analysis, analysis, and post-analysis stages. Ensuring diagnostic precision is vital for supporting a treating physician which helps in decreasing the patient mortality and morbidity significantly. Quality management in the cytopathology laboratory presents greater challenges compared to other laboratory disciplines, primarily due to the subjective nature of descriptive reports and variability in interpretations, compounded by the absence of numerical data. The varied terminologies used in cytology further complicate the implementation of quality control practices [1]. In the current era of automated laboratory practices, pre-analytical tests significantly influence the occurrence of preventable, irrelevant, and clinically noncontributory test outcomes [2]. The pre-analytical stage of quality control encompasses all procedures conducted in the laboratory before the analysis or reporting of the appropriate investigation. Pre-analytical is known to be the most vulnerable stage in the diagnostic process in a medical laboratory, accounting for 49% to 68% of flaws [3]. This study examines the frequency of pre-analytical errors leading to sample rejections during one year in a tertiary care hospital.

## Materials and methods

This study was conducted at the Cytology Laboratory of our hospital from January 2023 to December 2023, after getting proper approval from the Institutional Review Board (IRB approval number 101/02/2024/PG/SRB/SMCH). It is a retrospective analytical study, and the test samples from patients of the outpatient (OP) department, inpatient (IP) department, and community health outreach program facilities, especially Papanicolaou (PAP) smears were analyzed. The study population included patients undergoing cytological examination, and healthcare professionals involved in the pre-analytical phase of cytological specimen handling, including clinicians, nurses, laboratory technicians, pathologists, and the staff members responsible for sample collection, transportation, processing, and storage within the cytology laboratory. A total of 5,412 samples received in the cytology laboratory were analyzed during the study period, out of which, 3,758 (69.4%) were outpatient samples and 1,654 (30.6%) were inpatient samples. A total of 225 (4.16%) samples were repeated, of which 132 (2.44%) were outpatient samples and 93 (1.7%) were inpatient samples. This sample size provided a substantial dataset for the study. The inclusion criteria were the test samples sent specifically for cytological analysis. The samples sent for biochemical or microbiological examination were excluded. The cytology samples collected from various anatomical sites include fine needle aspiration specimens from palpable lesions such as thyroid swelling, breast lump, lymph node swelling, and soft tissue lesions obtained using aseptic precautions either directly or under ultrasound guidance. The fluid cytology samples were bronchoalveolar lavage, bronchial washings, sputum samples, urine, and effusions (pleural fluid, pericardial fluid, and ascitic fluid) collected using appropriate techniques such as bronchoscopy, thoracentesis, and paracentesis. The gynecological samples were cervical (PAP smears) and vaginal cytology specimens collected using standard protocols. Informed consent was obtained from the patients before undergoing any cytological procedures in compliance with institutional ethical guidelines and protocols. After collection, the samples were transported to the cytology sample processing area in appropriate preservative solutions or transport media suitable for cytological examination to preserve cellular integrity during transport. All the relevant data regarding the samples like the nature of the sample, received date and time, the number of slides in case of fine-needle aspiration, volume, and color of the fluids, and appropriate test request forms were collected and recorded in the corresponding cytology registers. The relevant clinical details and investigation details regarding any case were taken from the test request forms. Any errors that occurred till these processes were promptly noted in the pre-analytical registers. The fine-needle aspiration specimens were smeared onto the glass slides immediately after collection and fixed using appropriate fixatives (like alcohol-based fixatives). The fluid and gynecological samples were processed to concentrate cellular material by centrifugation and then smeared and fixed. The prepared smears were stained using standard cytological staining protocols with hematoxylin and eosin stain and PAP stain to facilitate the visualization of cellular morphology. Finally, the slides were examined under a light microscope to assess cellular morphology, nuclear features, cytoplasmic characteristics, and architectural patterns. The data for our study was taken from the registers and monthly statistics maintained in our laboratory and tabulated in the Excel sheet. The parameters measured were the pre-analytical errors (like specimen labeling errors, transportation delays, inadequate sample volume, sample contamination, specimen identification discrepancies, and incorrect specimen collection techniques), turnaround times (time taken from sample collection to reporting cytological results), staff performance, and sample quality. The frequency of sample distribution and rejected samples were calculated and the results were correlated. Based on the results and the aim of enhancing accurate results and efficient turnaround times, some strategies could be fostered like staff training and education, standardized protocols, quality control measures, specimen tracking systems, and continuous improvement initiatives.

## Results

A total of 5,412 samples received in the Cytology Laboratory were analyzed during the study period. This sample size provided a substantial dataset for the study. Out of which, 3,758 (69.4%) were outpatient samples and 1,654 (30.6%) were inpatient samples. This distribution reflected the typical distribution of samples in many healthcare settings, where outpatient samples tend to be more prevalent. A total of 225 (4.16%) samples were repeated, of which 132 (2.44%) were outpatient samples and 93 (1.7%) were inpatient samples. This indicated that higher rate of pre-analytical errors with outpatient sample collection, handling, or processing compared to inpatient samples. The majority of the repeated samples were PAP smears (2,352, 43.5%), followed by fluid cytology (1,008, 18.6%) and ultrasound-guided FNAC (984, 18.2%). This breakdown provided insights into the specific types of cytology tests that are more prone to errors requiring repetition. Also, 27 (0.5%) samples were rejected, of which the majority were PAP smears and thyroid FNAC. The rejection of samples indicated potential errors that rendered them inefficient for analysis, significantly impacting patient care and diagnostic outcomes. The values are summarized in Table 1.

**Table 1:** Distribution of cytology samples.

Serial number	Nature of sample	Number of samples			Number of samples repeated			Number of samples rejected
		Inpatient	Outpatient	Total	Inpatient	Outpatient	Total	
1.	Fine-needle aspiration cytology of thyroid	25	263	288	06	22	28	06
2.	Fine-needle aspiration cytology of lymph nodes	07	185	192	09	07	16	01
3.	Fine-needle aspiration cytology of the breast	11	241	252	05	17	22	02
4.	Fine-needle aspiration cytology of soft tissue	02	106	108	02	09	11	0
5.	Fine-needle aspiration cytology of the salivary gland	08	136	144	03	06	09	01
6.	Ultrasound-guided fine-needle aspiration cytology	232	752	984	20	28	48	02
7.	Vaginal cytology - Papanicolaou smear	367	1,985	2,352	24	29	53	13
8.	Fluid cytology (like ascitic, pleural, and bronchial)	997	11	1,008	22	10	32	02
9.	Exfoliative cytology (like sputum and skin)	05	79	84	02	04	06	0

The pre-analytical flaws identified as the reasons for rejection included insufficient samples, inconsistencies in registration, spilled samples, incorrect receptacles, sample clots, damaged slides, and incomplete or dirty forms for test requests, as shown in Table 2.

**Table 2:** Distribution of samples rejected.

Serial number	Causes of rejection	Inpatient samples	Outpatient samples	Total	Percentage
1.	Insufficient sample	1	16	17	63%
2.	Inconsistency in the registration	0	03	03	11.1%
3.	Spilled sample	0	02	02	7.4%
4.	Broken slides	01	01	02	7.4%
5.	Soiled test request forms	01	00	01	3.7%
6.	Wrong containers	0	01	01	3.7%
7.	Clots in the sample	01	0	01	3.7%

**Figure 1:**
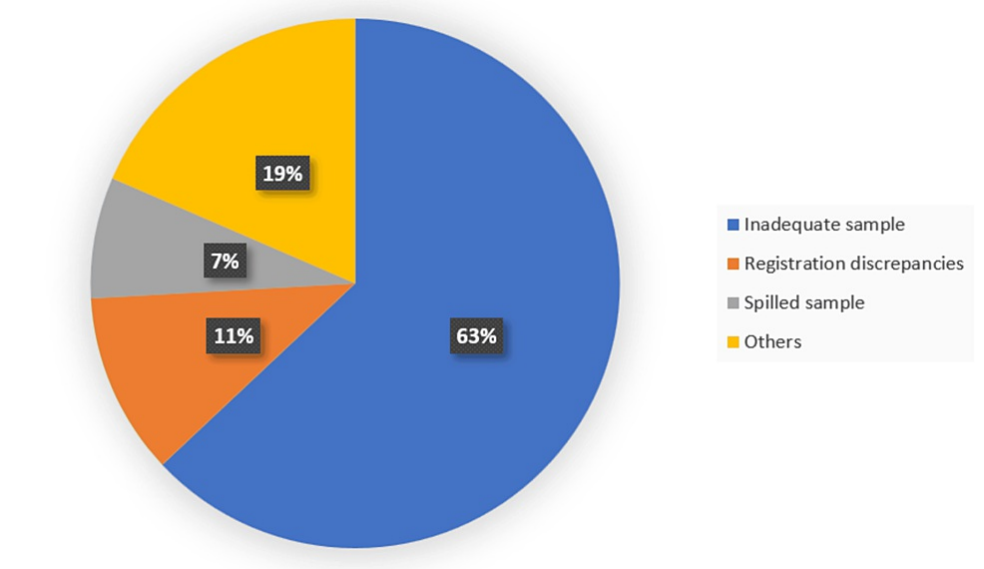
Frequency of rejected samples

Out of 27 rejected samples, the major causes for rejections were ascribed to insufficient sample (17, 63%), with inconsistencies in the registration process (3, 11.1%) and sample spillage (2, 7.4%), as shown in Figure 1.

Overall, the results highlighted the prevalence of pre-analytical errors in our cytology laboratory, with a notable proportion of samples requiring repetition or being rejected. The higher repetition rate among outpatient samples suggested the need for targeted interventions to improve pre-analytical processes, particularly in outpatient settings, to enhance the accuracy and efficiency of cytology services in our hospital. The specific types of tests more prone to errors helped in the development of targeted quality improvement initiatives. Additionally based on the results, some strategies could be fostered to enhance accurate results and efficient turnaround times in our cytology laboratory. Implementing comprehensive training programs for laboratory staff involved in sample collection, handling, processing, and analysis which include training on standardized protocols, proper specimen labeling techniques, and adherence to quality control measures. Develop and implement standardized protocols for specimen collection, handling, transportation, and processing to minimize errors and ensure consistency in laboratory practices which include establishing clear guidelines for each type of cytology test to reduce variability and improve accuracy.

## Discussion

Cytological analysis serves as a crucial diagnostic tool for physicians in both treatment initiation and disease classification. For instance, pivotal cytological reports such as those indicating unsuspected malignancy enable treating clinicians to promptly pursue appropriate treatment options [3]. Furthermore, any healthcare facility with limited assets can collect samples for cytology studies other than guided FNAC because guided FNAC requires less room and expensive equipment [4]. Pre-analytical, analytical, and post-analytical processes are the three key factors that determine the dependability and precision of cytological test results. In the current era of automated laboratory practices, significant strides have been made in minimizing errors during the analytical and post-analytical phases. However, pre-analytical errors persist as a major concern, highlighting the ongoing challenge of ensuring accuracy and reliability in diagnostic testing processes [2]. In the present study, the overall number of errors found in our cytology lab over one year accounted for 4.4% (238) of all investigations. This was statistically comparable to the studies conducted by Muthukrishnan et al. where total errors constituted 12.7% and Chandra et al. where total errors were observed to be 7.1% [1,2]. Also, in our study, the most accounted reason for rejection was the inadequate sample, which was statistically concordant with the study by Muthukrishnan et al. followed by discrepancies in the registration which is also concordant with the study by Chandra et al. where the reason was found to be incomplete or wrongly filled test request forms [1,2]. Most laboratory errors happen during the pre-analytical stage of the entire laboratory testing procedure. According to studies, preanalytical flaws, which can range from 31.6% to 75%, were the most common in the laboratory. This pre-analytical phase can be further classified into two distinct segments: the *Pre*-pre-analytical phase, constituting approximately 46% to 68% of errors, where the errors occur from the samples before reaching the laboratory and the *true* or *conventional* pre-analytical phase represents approximately 3% to 5% of errors, where the errors occur at the laboratory [5]. For a clinically relevant test report, cytological analysis needs an appropriate amount of epithelial cells of thyroid follicles in the thyroid FNAC and a certain quantity of epithelial cells of ducts in the FNAC of the breast and salivary glands. The primary cause of redo and sample rejection, according to this study, is inadequacy of the sample, which means not enough cells in FNAC, constituting 63% of cases. A precise needle biopsy produces a sufficient sample for the intended diagnosis. In broad terms, the optimal adequate biopsy offers a sample that is indicative of the lesion and contains the most legitimate diagnostic data in the quickest possible time, while also posing the least amount of stress to the patient, the laboratory technician, and the healthcare system overall. In terms of concept, needle biopsies are categorized as either insufficient, slightly adequate, or completely adequate for the desired diagnostic use. The qualities that make up the perfect needle biopsy sample include when it indicates the exact location of the lesion, when it includes cells and the content of cell components for the desired assessment when it includes cells and content of cell components that are indicative of the lesion, and when it contains particular tissue of the lesion rather than surrounding normal tissue, maximizing the diagnostic yield [6]. A cytology specimen containing peripheral blood cells may have a distorted morphology of cells, necessitating a repeat sample or inappropriate assessment, or it may indicate that the sample should be disregarded [7]. It constitutes 3.7% of the rejected samples in our investigation, underscoring its impact on sample quality and diagnostic assessment. FNAC of soft tissue lesions typically yields lower cellularity and necessitates an intrusive biopsy. The creation of specimen collection guidelines, instruction on specimen collection methods such as avoiding aspiration for thyroid cytology, coordination among reporting pathologists and clinicians collecting specimens as an outpatient procedure, and regular audits can reduce the need for retakes and sample rejection and achieve ideal turnaround time in the lab per the hospital's requirements [8]. Another significant factor leading to sample rejection and extended turnaround time is the occurrence of sample spillage, resulting in soiled test request forms that contain crucial clinical details such as the last menstrual period and the site of primary collection necessary for cytological analysis [9]. This issue accounts for 11.1% of rejected samples in the current study. Implementing training sessions on proper sealing techniques for sample containers holding bodily fluids, utilizing leak-proof sealing materials, and conducting regular audits of corrective and preventive measures can markedly decrease the rejection rate in ascitic and peritoneal fluid cytology analyses. The study's next noteworthy rejection criterion is the presence of broken slides of vaginal cytology, which are obtained as a component of clinical outreach initiatives. Our research identifies 7.4% of rejections attributed to broken slides. This pre-analytical error can be substantially mitigated through training initiatives and the provision of appropriate packing materials. Using improper sample containers can compromise valuable samples like synovial fluid, while an excess of peripheral blood can lead to sample clotting, rendering it unsuitable for processing [1,10]. In our study, 3.7% of rejections were attributed to the use of incorrect containers. These mistakes in the laboratory will be greatly decreased with proper training and auditing.

Limitations

The major limitations of this study include a smaller sample size, thereby limiting its scope and potentially affecting the generalizability of the findings to the broader population. Limited to a single-center study, conducting the research in only one tertiary care hospital restricts the diversity of cytology practices and sample characteristics, thereby diminishing the external validity of the findings. The study was prone to selection bias, as it had only included cytology samples from patients who presented to the hospital, excluding those from other healthcare settings or those who did not seek medical care. Regarding the study design, being retrospective led to potential information bias or missing data, impacting the accuracy and completeness of the findings. Additionally, the problem of limited variables arose as the study focused only on specific pre-analytical errors, potentially overlooking other factors that could have contributed to cytology sample quality. Context specificity was evident, as the findings were specific to the practices and resources available in a particular hospital setting in South India, limiting generalizability to other regions or healthcare systems.

## Conclusions

Pre-analytical, analytical, and post-analytical processes are the three key factors that determine the dependability and precision of cytological test results. Detecting critical warnings such as the positivity of malignancy underscores the paramount importance of result accuracy. Implementing good laboratory practices and conducting both external and internal audits can reduce the frequency of preventable errors in a cytological laboratory, thereby ensuring enhanced precision and expedited outputs. The reliability and accuracy of cytological test results hinge on three crucial variables: pre-analytical, analytical, and post-analytical processes. The detection of critical alerts such as the presence of malignancy underscores the paramount importance of result accuracy. Implementing good laboratory practices and conducting both external and internal audits can reduce the frequency of preventable errors in a cytology laboratory, thereby ensuring enhanced accuracy and expedited results. Our study highlights the significance of evaluating pre-analytical flaws in the cytology laboratory and underscores the need for implementing strategies to enhance result accuracy and turnaround times. By identifying and addressing pre-analytical challenges such as sample spillage, broken slides, and inappropriate sample containers, we can improve the reliability of cytological test results. Moreover, fostering a culture of continuous improvement through training initiatives and regular audits will contribute to the overall efficiency of the laboratory, ensuring timely and accurate diagnoses for patients in need of cytological analyses.
